# Imaging in Vasculitis

**DOI:** 10.1007/s11926-020-00915-6

**Published:** 2020-06-19

**Authors:** Konstanze Viktoria Guggenberger, Thorsten Alexander Bley

**Affiliations:** Department of Diagnostic and Interventional Radiology, Faculty of Medicine, University Hospital Wuerzburg, University of Wuerzburg, Oberduerrbacher Straße 6, 97080 Wuerzburg, Germany

**Keywords:** Vasculitis, Large vessel vasculitides (LVV), Giant cell arteritis (GCA), Imaging, Magnetic resonance imaging (MRI), EULAR guidelines

## Abstract

**Purpose of Review:**

Vasculitides are characterized by mostly autoimmunologically induced inflammatory processes of vascular structures. They have various clinical and radiologic appearances. Early diagnosis and reliable monitoring are indispensable for adequate therapy to prevent potentially serious complications. Imaging, in addition to laboratory tests and physical examination, constitutes a key component in assessing disease extent and activity. This review presents current standards and some typical findings in the context of imaging in vasculitis with particular attention to large vessel vasculitides.

**Recent Findings:**

Recently, imaging has gained importance in the management of vasculitis, especially regarding large vessel vasculitides (LVV). Recently, EULAR (European League Against Rheumatism) has launched its recommendations concerning the diagnosis of LVVs. Imaging is recommended as the preferred complement to clinical examination. Color-coded duplex sonography is considered the first choice imaging test in suspected giant cell arteritis, and magnetic resonance imaging is considered the first choice in suspected Takayasu’s arteritis.

**Summary:**

Due to diversity of clinical and radiologic presentations, diagnosis and therapy monitoring of vasculitides may constitute a challenge. As a result of ongoing technological progress, a variety of non-invasive imaging modalities now play an elemental role in the interdisciplinary management of vasculitic diseases.

## Introduction

Vasculitis is a collective term for a heterogeneous group of inflammatory vascular diseases. Clinical and radiologic appearance is variable, depending decisively on the size, number, and location of the affected vessels. According to the revised Chapel-Hill-Consensus-Conference nomenclature of vasculitides (2012), vasculitides are subdivided into primary and secondary vasculitides [1]. Primary vasculitides are autonomous, mostly autoimmunologically induced pathologies belonging to the rheumatic diseases [1]. Secondary vasculitides occur in association with or as a result of other diseases or drugs [1]. Primary and secondary vasculitides are subclassified according to the size of the affected vessels in small, medium-sized, and large vessel vasculitides or vasculitides with variable vessel sizes [1].

### Imaging in Vasculitides

In many forms of vasculitis, morphologic changes in the context of the disease can be visualized by radiologic imaging methods, either directly by visualizing the vessel lesions or indirectly by visualizing the consequences of vessel inflammation in the affected organ. The principles of imaging of primary and secondary vasculitides do not differ—the choice of the adequate imaging modality depends on the size and localization of the affected vessels. A number of different imaging techniques are available for visualizing direct and indirect signs of vessel inflammation in LVV. Vessel lesions in small vessel vasculitides are usually below the radiologic detection limits. However, indirect signs of small vessel vasculitides may be revealed by imaging and usually display disease activity and extent [2, 3]. Additionally, imaging can help in the selection of the best biopsy point [2, 3]. In the following, the most common vasculitides and their appearance on imaging are presented, with a focus on LVV.

Table 1 summarizes appropriate technical parameters for the most common imaging modalities in vasculitis.

**Table 1:** Recommendations regarding technical parameters for the most common imaging modalities in LVV, adapted and modified from *15*

Imaging modality and indication	Technical parameters
Ultrasound	- Supra-aortic arteries: linear probes - Ascending aorta and aortic arch: sector or convex probes - Abdominal aorta: convex probes - Temporal arteries: B-mode frequency >/= 15 MHz - Extracranial supra-aortic arteries: B-mode frequency for extracranial 7–15 MHz - Temporal arteries: Doppler frequencies 7–12 MHz - Extracranial supra-aortic arteries: Doppler frequencies 4–8 MHz - Angle between sound waves and artery </= 60° - Focus at the level of the artery - Color Doppler preferred over power Doppler mode
CT	- Multislice CT scanner preferred - Collimation 0,6 mm, tube voltage 120 kV - Tube current time product (mAs) determined by automatic dose modulation - Reconstruction slice thickness 0.5–1.0 mm - Bodyweight adapted injection of 60–120 mL of non-ionic iodinated contrast agent (>/= 350 mg/mL) using a power injector (>/= 4 mL/s) - Arterial phase: bolus tracking method (threshold of 100 HU), ECG triggering - Venous phase: acquisition 50 s after finishing the arterial phase
MRI	Cranial technique: - 1,5, preferentially 3,0 T MRI scanner, minimum 8 channel head-coil - T1-weighted spin echo, gadolinium contrast-enhanced, fat-suppressed, high-resolution (in plane << 1 mm^2^, e.g., 195 × 260 μm, slice thickness 3 mm, repetition time (TR)/echo time (TE) 500/22 ms) - T2-weighted turbo spin echo (TSE), non-contrast-enhanced imaging (TR/TE 9000/143 ms) significantly less sensitive - Transversal slices angulated parallel to skull base Body technique: - 1,5, preferentially 3,0 T MRI scanner, minimum 8 channel head and neck coil, 16 channel body coil - MR angiography of aorta and major branches from carotid bifurcation to iliac arteries in coronal acquisition to include axillary and brachial arteries → Detection of vessel lumen (stenosis, occlusion, aneurysm) - T1-weighted, fat-suppressed, contrast-enhanced, black blood imaging (e.g., navigated 3D TSE, spatial resolution 1.2 × 1.3 × 2 mm^3^, TR/TE 1000/35 ms) → Assessment of mural inflammation - T2-weighted TSE imaging for edema detection in mural inflammation less sensitive and more vulnerable to artifacts
FDG-PET-CT	- Hybrid PET combined with low-dose CT - Blood glucose levels: preferred < 7 mmol/L (126 mg/dL), < 10 mmol/L (180 mg/dL) acceptable - Interval between FDG infusion and image acquisition at least 60 min, preferably 90 min - Position of patient is supine, position of the arms: arms down - Body part to include from top of head to at least mid-thigh, preferably to below the knees - Scoring FDG uptake, qualitative visual grading; if results unclear, comparison with the liver background (grading 0–3)

#### Small Vessel Vasculitides

Granulomatosis with polyangiitis (GPA, formerly known as Wegener’s granulomatosis) is an autoimmunologically induced, necrotizing and granulomatous inflammatory disease of small and medium-sized vessels, mostly affecting the upper respiratory tract and the kidneys, with the nasal cavity, the paranasal sinus, and the mastoid cells as predilection sites [4]. In addition to laboratory and histopathologic results, imaging plays an important role in the evaluation of disease activity and extent. MRI is the modality of choice for the evaluation of affected soft tissue. Computed tomography (CT) is superior in the visualization of changes in the osseous structures and the lungs. Typical radiologic findings in the framework of GPA comprise mucosal swelling, bone erosion, and bone regeneration. Mucosal swelling in the framework of GPA most often affects the maxillary sinus, while osseous changes most often occur in the ethmoidal cells. Differentiation between vasculitic lesions, chronic inflammatory, and malignant processes, as well as lymphoma, may be difficult [2, 4, 5]. Mucosal swelling in the context of GPA in the stage of advanced granulomatous transformation often shows a nodular configuration with a hypointense signal in T2-weighted MRI-sequences in contrast to the typically iso- to hyperintense signal of inflammatory or malignant lesions [2, 4, 5].

Another predilection site of GPA is the lower respiratory tract, especially the lung. Both inflammatory alveolar infiltrates and granulomatous changes of the lung structure, particularly pulmonary nodules, are possible manifestations [6, 7]. Whereas the pulmonary nodules are mostly asymptomatic, the infiltrates can develop to pulmonary hemorrhage and in some cases proceed to the more serious, potentially deadly, pulmonary-renal syndrome [3]. The method of choice for visualization of pulmonary changes is CT.

#### Medium-Sized Vessel Vasculitides

##### Polyarteritis Nodosa

Polyarteritis nodosa (PAN) is an autoimmunologically mediated systemic necrotizing form of vasculitis, affecting small and medium-sized vessels. Inflammatory vessel changes in the context of PAN most often occur in the medium-sized vessels of the heart, kidneys, nervous system, gastrointestinal tract, and musculoskeletal system [8]. Usually, inflammation affects all layers of the vessel wall, possibly resulting in stenosis, occlusion, or aneurysms. The appropriate imaging method depends on the affected organ system [8]. Echocardiography, CT-angiography, and, in case of interventional therapy, catheter angiography are commonly used techniques in evaluation of the heart and coronary arteries. CT is typically used for the visualization of incurred complications, such as bleedings or necrosis in all organ systems. Depending on the size of the affected vessels, high-resolution CT angiography or, the more sensitive, catheter angiography is able to reveal the respective vessel lesions themselves.

##### Kawasaki Disease

Kawasaki disease is a necrotizing inflammation of small and medium-sized vessels throughout the body, associated with fever and systemic inflammatory reaction of many organs. The inflammatory processes might result in serious complications, particularly in the case of cardiac involvement, with cardiac infarctions and myocarditis with possible involvement of the heart valves, as well as aneurysms and the potential risk of their rupture or thrombosis. Echocardiography is the method of choice for the evaluation of coronary arteries, heart valves, and heart function [9]. Despite its superior sensitivity, especially in cases of distant coronary aneurysms, catheter angiography needs to be used responsibly because of its invasiveness and radiation exposure.

#### Large Vessel Vasculitides (LVV) and EULAR Guidelines

Giant cell arteritis (GCA) and Takayasu’s arteritis (TA) are subsumed under the term LVV, differing mainly in terms of epidemiological conditions, involvement pattern, and clinical appearance. LVV are autoimmune diseases characterized by a granulomatous inflammation of large and medium-sized vessel walls. Identifying patients with LVV may be challenging, as they often present with a combination of nonspecific clinical symptoms and a systemic inflammatory profile on laboratory results. Temporal artery biopsy is still considered the “gold standard” in diagnosing the cranial form of GCA [10–12]. Classification criteria for GCA of the American College of Rheumatology include various clinical points and histopathological findings of the superficial temporal artery, but there are no imaging criteria [10]. However, due to ongoing technological progress and the combination of good diagnostic reliability and low invasiveness, imaging has gained significantly in importance in the management of LVV. The EULAR has recently launched non-binding recommendations for the use of imaging in the process of diagnosing and monitoring of LVV [13••]. According to the EULAR guidelines, an early imaging test is considered the first and preferred complement to clinical and laboratory criteria in patients with suspected LVV [13••]. In the context of the individual interpretation of the findings, it is important to consider that sensitivity of imaging tests decreases significantly a few days after initiation of corticosteroid therapy [14]. Therefore, imaging tests should be performed as soon as possible, ideally prior to therapy initiation. However, initiation of therapy must not be delayed due to the unavailability of adequate imaging methods. In cases of suspected LVV and the unavailability in the foreseeable future of adequate imaging methods or expertise, other diagnostic tests should be performed for clarification. In cases of high clinical suspicion, therapy should be initiated despite an incomplete diagnostic process. Table 1 summarizes recommendations regarding technical parameters for the most common imaging modalities in LVV.

##### Giant Cell Arteritis (GCA)

**Fig. 1:**
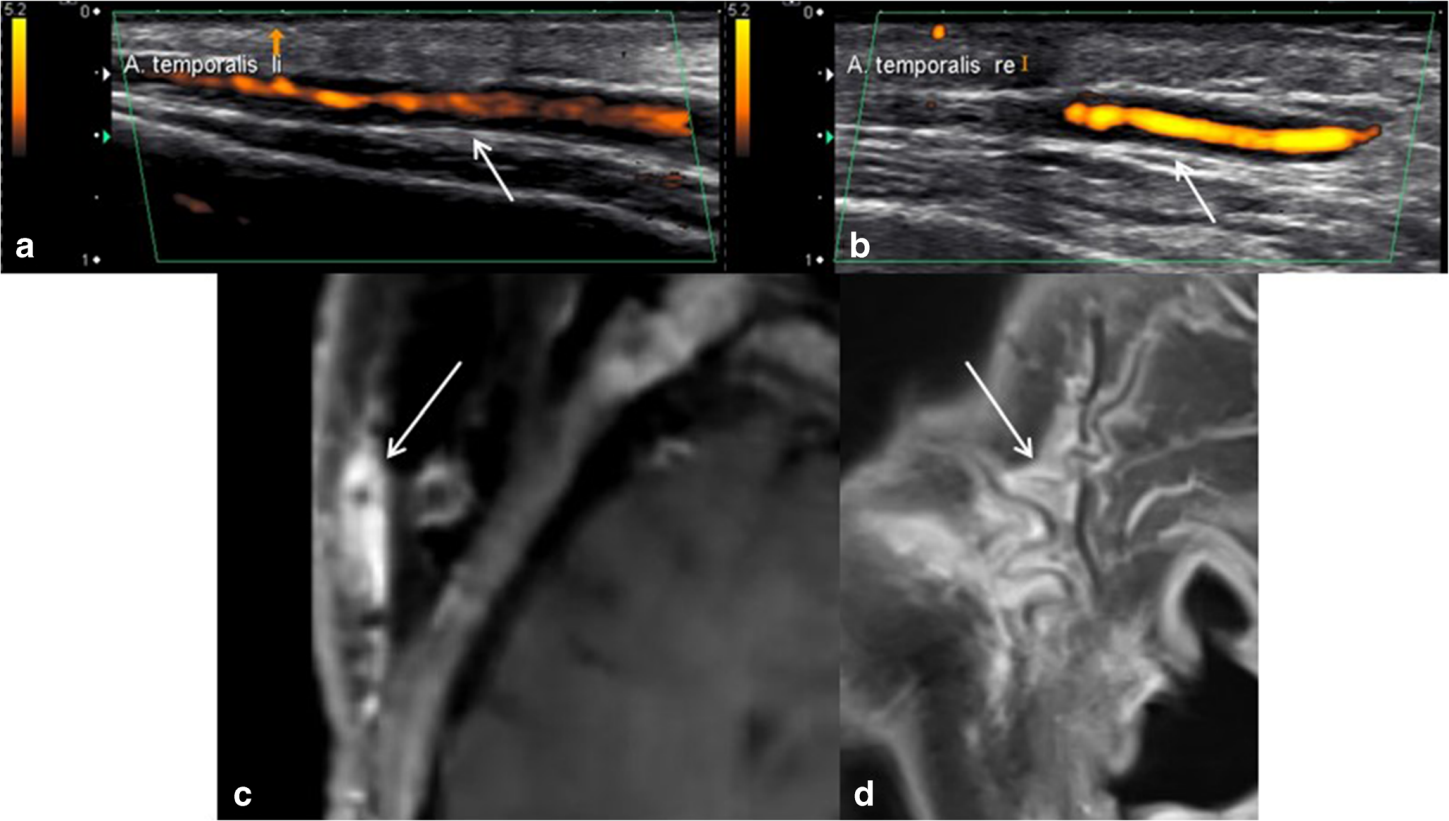
Typical imaging findings in GCA: ultrasound under compression shows a non-compressible long-segmental hypoechoic “Halo” sign around both temporal arteries, representing inflammatory vessel wall edema (**a**, **b**, white arrows). T1-weighted contrast-enhanced MRI visualizes the correlating concentric vessel wall enhancement of the superficial temporal arteries and the extended inflammatory reaction of the surrounding tissue (**c**, **d**, white arrows)

**Fig. 2:**
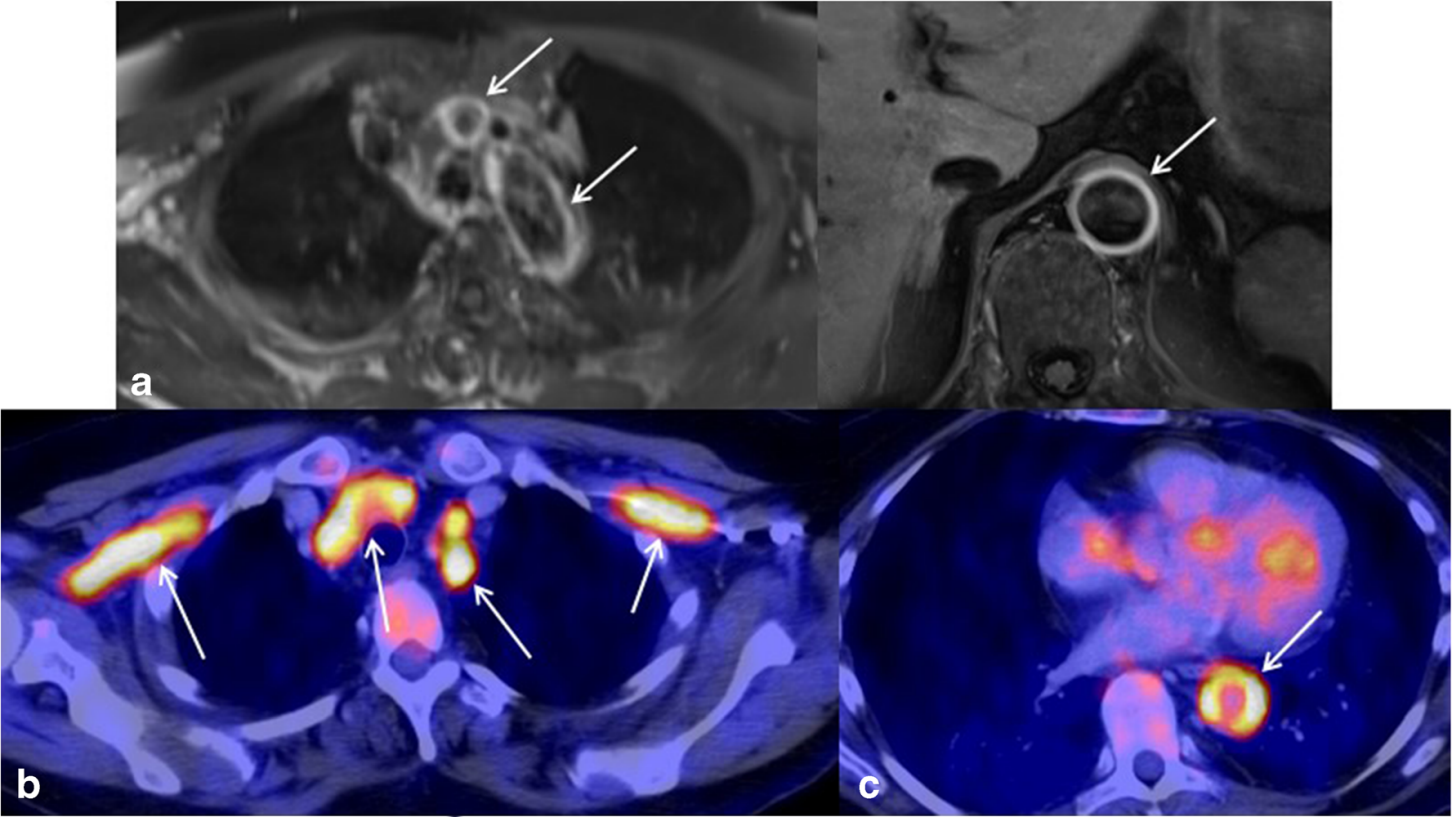
Characteristic findings in GCA with involvement of the aorta and first major branches: T1-weighted MRI shows smooth concentric vessel wall thickening and enhancement of the thoracic and abdominal aorta and the brachiocephalic trunk (**a**, **b**, white arrows). FDG-PET-CT reveals a correlating significant tracer uptake of the inflamed vessel walls along the thoracic aorta (**d**, white arrow) as well as along the supra-aortic branches including the bilateral axillary artery (**c**, arrows)

GCA is a systemic autoimmune disease, usually occurring in elderly people (> 50 years), often associated with polymyalgia rheumatica. It typically affects the supra-aortic arteries, including the vertebral, subclavian/axillary, superficial temporal, and occipital arteries. Against the background of possible severe complications, especially threatening blindness because of insufficient blood circulation in the optic disc in cases of involvement of the posterior ciliary arteries, timely and reliable diagnosis is important. Temporal artery biopsy is considered the gold standard for diagnostic confirmation despite its invasiveness. However, imaging has significantly gained in importance recently, being invasive and having good diagnostic reliability. Besides clinical examination and laboratory tests, EULAR recommends color-coded duplex sonography (CCDS) as the first imaging test for suspected GCA. Sonography is non-invasive, fast, available, reliable, and inexpensive. Pathognomonic sonography findings in the cranial form of GCA include the so-called “Halo” sign, a non-compressible hypoechoic, in axial orientation concentric vessel wall thickening around the superficial temporal artery > 340–420 μm, depending on the specific superficial cranial artery segment [15••, 16•], corresponding to a vessel wall edema. With modern ultrasound transducers, image resolution of 0.1 mm can be achieved for superficial cranial arteries [17•]. Alterations of the arteries’ flow curves in cases of hemodynamically relevant stenoses or even vessel occlusion may also be observed. Measurement of the thickness of the intima and media taken together (intima-media thickness) can help distinguish vasculitic from healthy arteries with a relatively high predictive value [16•]. Contrast-enhanced ultrasound may help confirm the diagnosis by visualizing hyperemia and hypervascularization of vessel walls, typical characteristics of a florid inflammatory process [18]. In cases of inconclusive results in CCDS, contrast-enhanced MR imaging is recommended according to the EULAR guidelines for further clarification [13••]. CCDS and high-resolution MRI have been demonstrated to visualize inflammatory vessel wall changes with comparable values regarding sensitivity and specificity [19•, 20]. Typical MRI findings in GCA comprise circumferential thickening and contrast enhancement of the vessel’s wall, facultatively with consecutive narrowing of its lumen. Mural thickness > 600 μm serves as a cut-off value for a positive result [21]. The lower cut-off values in CCDS with > 340–420 μm as compared with MRI with > 600 μm are explained by the higher spatial resolution of CCDS which allows for more precise mural thickness measurements. Compared with the usually eccentric, irregular, and heterogeneous characteristics of degenerative atherosclerotic wall changes and stenosis, inflammatory mural thickening, edema and enhancement, and consecutive stenosis typically have a smooth and concentric character. Figure 1 shows typical imaging findings in a GCA patient with involvement of both superficial temporal arteries. Figure 2 shows typical imaging findings in a GCA patient with involvement of the thoracic and abdominal aorta as well as of the large supra-aortal arteries.

The combination of high clinical probability and a positive imaging test allows diagnosing GCA without any further tests [13••]. GCA may be considered unlikely in cases of low clinical suspicion combined with a negative imaging test [13••]. In cases of inconclusive results after clinical and laboratory examination and an imaging test, further steps should be taken to definitely confirm or exclude GCA [13••].

##### Takayasu’s Arteritis (TA)

**Fig. 3:**
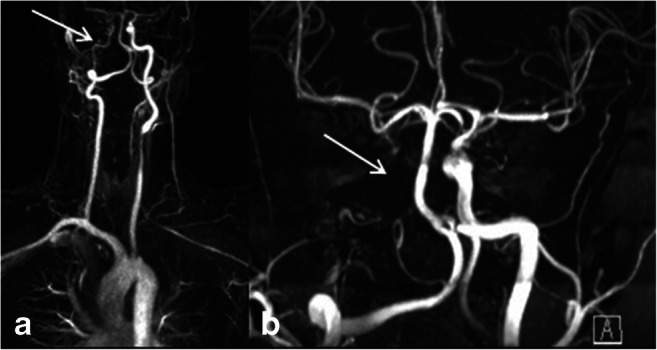
Imaging findings in a patient with Takayasu’s arteritis: Contrast-enhanced MRA shows a long segmental occlusion of the right carotid artery with a filiform contrasting of the right internal carotid artery (**a**, white arrow)

TA is an autoimmune-mediated granulomatous inflammation of the aorta and its major branches, mostly affecting relatively young people, < 50 years of age at disease onset. The granulomatous inflammation particularly affects the medium layer of the vessel wall. Analogous to the morphologic changes in GCA, typical radiologic findings in TA include thickening and contrast enhancement of the inflamed vessel wall, resulting in narrowing of the affected vessel’s lumen. Stenosis and occlusion with the risk of ischemic (brain) injuries, and less often aneurysms and artery dissection, are known complications of chronic TA. Figure 3 shows a patient with a known Takayasu’s arteritis with chronic occlusion of the right common carotid artery.

According to the EULAR guidelines, MRI is the imaging test of choice to capture disease extent and activity in TA [13••]. CT angiography and 18F-fluorodeoxyglucose positron emission tomography combined with computed tomography (FDG-PET-CT) and/ or CCDS may be used as equivalent options [13••]. However, as a result of the anatomic localization of the potentially affected vessels, such as the thoracic aorta, CCDS usually plays a less important role in the context of TA [13••].

In both GCA and TA, involvement patterns of vasculitic changes are highly variable. Cross-sectional imaging techniques, by means of a relatively wide scan range, enable assessment of the majority of the body’s vasculature and allow assessment of vessel walls and lumens, as well as the surrounding tissue simultaneously in a single examination, and are therefore well suited for defining the disease extent in LVV. FDG-PET-CT enables visualization of vessel wall hypermetabolism, a characteristic, yet nonspecific, feature of vasculitis. Visual tracer uptake of the vessel wall higher than in the liver is suggestive for vasculitis in LVV [22]. A linear or segmental pattern of tracer uptake in the aorta and its branches is characteristic for GCA [23]. Due to its invasiveness and the lack of adequate vessel wall imaging, conventional angiography has been mostly replaced by less invasive imaging techniques [13••].

Modality and frequency of repeat imaging for long-term monitoring in LVV should be adjusted according to individual circumstances. However, routine imaging is not recommended for patients in clinical and laboratory remission [13••].

#### Central Nervous System (CNS) Vasculitis

**Fig. 4:**
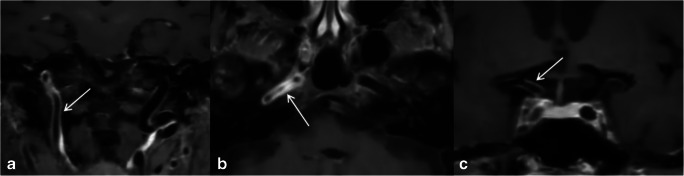
GCA patient with intracranial vasculitic involvement: T1-weighted MRI reveals long-segmental concentric wall thickening and enhancement of the right ICA in its extra- and intradural course (**a**–**c**, white arrows)

The term CNS vasculitis comprises all vasculitic changes of the arteries of the circle of Willis, including primary, isolated CNS vasculitides and secondary CNS vasculitis, occurring in the framework of systemic vasculitis or other diseases, especially autoimmune and infectious diseases, and in the context of organ rejection after organ transplantation and as a result of medication or drug intake. Radiologic findings in CNS vasculitis can be divided into primary and secondary signs [24]. Primary signs include mural inflammatory changes, namely, circular wall thickening and contrast enhancement and optional stenosis and, particularly, in case of chronicity, aneurysms. Figure 4 shows typical imaging findings in a patient with CNS vasculitis with vasculitic affection of the large intracranial arteries. Typical findings in vasculitis of small and medium-sized vessels are luminal stenosis alternating with dilatation, termed the “beading sign” [25]. Secondary signs comprise structural damage of brain tissue as a result of the ongoing inflammatory process, particularly multiple small, microvascular infarctions in different vascular territories and of different ages, as well as microbleeds, subarachnoid hemorrhage, or hemorrhagic infarctions. Contrast-enhanced MRI combined with magnetic resonance angiography is the method of choice in cases of suspected CNS vasculitis. Both luminal and mural lesions as well as potential structural brain damage can be visualized in the same examination. Time of flight and/or contrast-enhanced angiography may be used for visualization of the vessels’ lumens and potential stenoses, occlusion, or aneurysms. Usual sequences for evaluation of brain parenchyma, supplemented by diffusion-weighted imaging, susceptibility-weighted imaging (a sequence very sensitive to detect cerebral hemorrhage) and high-resolution, fat-suppressed contrast-enhanced sequences are suitable techniques for capturing parenchymal complications and inflammatory vessel wall enhancement. CT, combined with CT angio, is an alternative to MRI, as it can also visualize already incurred major changes of brain parenchyma and major vessel changes, such as relevant stenosis of large vessels, vessel occlusion, or aneurysms.

DSA (digital subtraction angiography) is more sensitive than MRI for the detection of vasculitic luminal changes of small- and medium-sized peripheral arteries. However, it does not visualize the vessel walls. Due to its relatively high invasiveness compared with MRI, as well as to its relatively low specificity regarding the underlying pathology of the luminal changes, DSA is reserved to diagnose cerebral vasculitis.

The most important differential diagnosis of primary and secondary CNS vasculitides include reversible cerebral vasoconstriction syndrome, intracranial atherosclerosis, fibromuscular dysplasia, Moyamoya disease, intravascular lymphoma, and radiogenic vasculopathies.

Table 2 lists the most common imaging modalities for the respective form of vasculitis as well as the respectively evaluable anatomic structures and typical imaging findings.

**Table 2:** Suitable imaging modality for the respective form of vasculitis with evaluable anatomic structures and typical imaging findings

Form of vasculitis	Suitable imaging modality	Evaluable structures/typical imaging findings
Granulomatosis with polyangiitis (GPA)	CT	Osseous structures (bone erosion, bone regeneration), mucosal swelling, pulmonary changes
	MRI	Mucosal swelling (typically nodular configuration with a hypointense T2-signal)
Polyarteritis nodosa	Echocardiography	Heart, heart valves, heart function
	CT/CT-angiography	Complications, bleedings, or necrosis in different organ systems, vessel lesions
	Catheter angiography	Vessel lesions (stenosis, occlusion, aneurysms)
Kawasaki disease	Echocardiography	Heart, heart valves, heart function
Giant cell arteritis (GCA)	Color-coded duplex sonography (CCDS)	“Halo” sign (non-compressible hypoechoic concentric vessel wall thickening around the superficial temporal artery > 340–420 μm, stenosis, occlusion)
	MRI/MRA	Vessel lesions, particularly of superficial temporal arteries (vessel wall thickening and enhancement, stenosis, occlusion) (intracranial) complications
Takayasu’s arteritis (TA)	MRI/MRA	Vessel lesions (vessel wall thickening and enhancement, stenosis, occlusion) intracranial complications
	CT-angiography/FDG-PET-CT	Vessel lesions (vessel wall thickening and enhancement, stenosis, occlusion), disease extent, and activity
CNS vasculitis	MRI/MRA	Vessel lesions (vessel wall thickening and enhancement, stenosis, occlusion), intracranial complications
	CT/ CT-angiography	Vessel lesions (vessel wall thickening and enhancement, stenosis, occlusion), intracranial complications
	Catheter angiography	Vessel lesions (stenosis, occlusion)

## Conclusion

Diagnosis and therapy monitoring of vasculitic diseases are often challenging because of the heterogeneity of subgroups and their variable clinical, laboratory, and radiologic manifestations; imaging often plays a key role. The choice of an adequate imaging method mainly depends on the size and localization of the affected vessels, as well as on the patient’s individual circumstances and the specific question. Depending on the affected vessels’ size, imaging is capable of visualizing the vessel lesion itself, helps in determining disease extent and activity, and defining already occurred complications. The EULAR has recently launched recommendations for the use of imaging in the diagnosis of large vessel vasculitides, with color-coded duplex sonography as the imaging modality of first choice in suspected giant cell arteritis and MRI as the imaging modality of first choice in case of Takayasu’s arteritis.
